# Supplementary figures and images for: Diversity Begets Diversity When Diet Drives Snake Venom Evolution, but Evenness Rather Than Richness Is What Counts
Source: Toxins (Basel). 2023 Mar 29;15(4):251. doi: 10.3390/toxins15040251 (PMC10142186; doi:10.3390/toxins15040251)

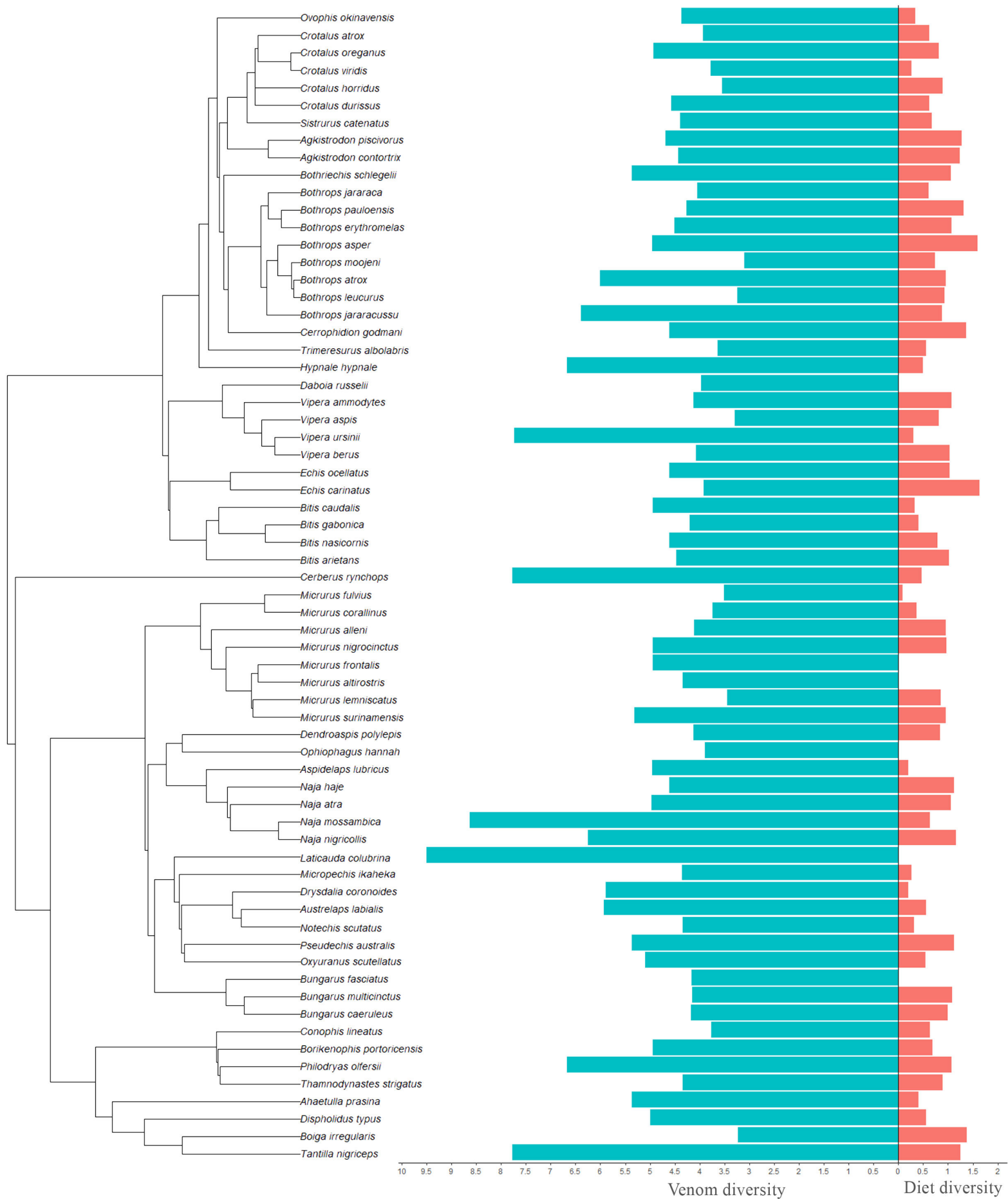

Supplement: Supplementary file 1 [file toxins-15-00251-s001.zip › toxins-2279427-supplementary.pdf]
